# EPIGENETIC AGE ACCELERATION AND IMMUNE CELL COUNT: A TWIN STUDY

**DOI:** 10.1093/geroni/igad104.2484

**Published:** 2023-12-21

**Authors:** Eric Penichet, Christopher Beam, Kelly Bakulski, Eric Turkheimer, Kristin Higdon, Lesa Ryan, Kendra Sikes, Deborah Davis

**Affiliations:** University of Southern California, Los Angeles, California, United States; University of Southern California, Los Angeles, California, United States; University of Michigan, Ann Arbor, Michigan, United States; University of Virginia, Charlottesville, Virginia, United States; Norton Children’s Research Institute, Louisville, Kentucky, United States; University of Louisville School of Medicine, Louisville, Kentucky, United States; Norton Healthcare, Louisville, Kentucky, United States; University of Louisville School of Medicine, Louisville, Kentucky, United States

## Abstract

Accelerated aging, as measured by epigenetic clocks, is associated with declines in immune system functioning. Investigating the causal basis of this association may provide mechanistic insight into the link between DNA methylation, on which epigenetic clocks are based, and age-related changes in immune cell composition. Using preliminary data from the Louisville Twin Study, this study examined the genetic and environmental sources of covariation between immune cell count (neutrophils, lymphocytes) and GrimAge Acceleration. GrimAge is a composite biomarker derived from 7 DNA methylation-based surrogates associated with mortality and smoking pack years. GrimAge Acceleration is the residual of GrimAge regressed onto chronological age. Participants consisted of 43 monozygotic twin pairs and 30 (18 same-sex) dizygotic twin pairs with a mean age of 54.5 years (sd = 5.27). Phenotypic analyses revealed that GrimAge Acceleration was significantly associated with neutrophil and lymphocyte count (r = 0.45 and -0.49, respectively). To examine the sources of covariation between these variables, biometric regression models were used to decompose the covariance between GrimAge Acceleration and neutrophils (and lymphocytes) into genetic and environmental components. Bivariate analyses revealed moderate, marginally significant genetic correlations between GrimAge Acceleration and immune cell count (rg = 0.32 and -0.38 for neutrophils and lymphocytes, respectively) and large, significant nonshared environmental correlations (re = .58 and -.54 for neutrophils and lymphocytes, respectively). Common genetic factors accounted for 39% of the phenotypic correlation in neutrophils and 43% in lymphocytes, whereas common nonshared environmental factors accounted for 61% and 57% of the correlation in these cell types, respectively.

